# Soil amendment with cow dung modifies the soil nutrition and microbiota to reduce the ginseng replanting problem

**DOI:** 10.3389/fpls.2023.1072216

**Published:** 2023-01-24

**Authors:** Setu Bazie Tagele, Ryeong-Hui Kim, Minsoo Jeong, Kyeongmo Lim, Da-Ryung Jung, Dokyung Lee, Wanro Kim, Jae-Ho Shin

**Affiliations:** ^1^ Department of Applied Biosciences, Kyungpook National University, Daegu, Republic of Korea; ^2^ NGS core facility, Kyungpook National University, Daegu, Republic of Korea; ^3^ Department of Integrative Biology, Kyungpook National University, Daegu, Republic of Korea

**Keywords:** co-occurrence network, functional prediction, ginseng, illumina miseq, replant failure, soil microbiome

## Abstract

Ginseng is a profitable crop worldwide; however, the ginseng replanting problem (GRP) is a major threat to its production. Soil amendment is a non-chemical method that is gaining popularity for alleviating continuous cropping obstacles, such as GRP. However, the impact of soil amendment with either cow dung or canola on GRP reduction and the associated soil microbiota remains unclear. In the present study, we evaluated the effect of soil amendment with cow dung, canola seed powder, and without amendment (control), on the survival of ginseng seedling transplants, the soil bacterial and fungal communities, and their associated metabolic functions. The results showed that cow dung increased ginseng seedling survival rate by 100 percent and had a remarkable positive effect on ginseng plant growth compared to control, whereas canola did not. Cow dung improved soil nutritional status in terms of pH, electrical conductivity, ${\text{NO}}_{3}^{-}$ , total carbon, total phosphorus, and available phosphorus. The amplicon sequencing results using Illumina MiSeq showed that canola had the strongest negative effect in reducing soil bacterial and fungal diversity. On the other hand, cow dung stimulated beneficial soil microbes, including *Bacillus*, *Rhodanobacter*, *Streptomyces*, and *Chaetomium*, while suppressing Acidobacteriota. Community-level physiological profiling analysis using Biolog Ecoplates containing 31 different carbon sources showed that cow dung soil had a different metabolic activity with higher utilization rates of carbohydrates and polymer carbon sources, mainly Tween 40 and beta-methyl-d-glucoside. These carbon sources were most highly associated with Bacillota. Furthermore, predicted ecological function analyses of bacterial and fungal communities showed that cow dung had a higher predicted function of fermentation and fewer functions related to plant pathogens and fungal parasites, signifying its potential to enhance soil suppressiveness. Co-occurrence network analysis based on random matrix theory (RMT) revealed that cow dung transformed the soil microbial network into a highly connected and complex network. This study is the first to report the alleviation of GRP using cow dung as a soil amendment, and the study contributes significantly to our understanding of how the soil microbiota and metabolic alterations *via* cow dung can aid in GRP alleviation.

## 1 Introduction

Ginseng (*Panax ginseng* C. A. Meyer) is an economically important medicinal plant in South Korea; however, its production is challenged by the ginseng replanting problem (GRP), which is caused by a variety of abiotic and biotic factors (Lee et al., 2015; Yang et al., 2015; Seo et al., 2019). Recent studies have documented that the changes in soil chemical properties, of which soil pH is the main factor, significantly contribute to GRP (Zhang et al., 2020). The soil microbiota plays a key role in determining plant health and productivity by channeling various crucial soil functionalities, including mineralization and plant disease control (Srivastava et al., 2022). Manipulating the soil microbiota is an effective way to alleviate GRP (Lee et al., 2015; Dong et al., 2018; Seo et al., 2019). Several agricultural practices can shape the soil microbial structure (Romdhane et al., 2022), which can result in either positive (Qi et al., 2020) or negative outcomes on plant health (Bziuk et al., 2021). For example, continuous cropping of ginseng affects the taxonomic and functional diversity of the soil microbial population and the potential pathogenic genera, increasing the risk of soil conduciveness (Zhang et al., 2020; Tong et al., 2021). On the other hand, various methods such as crop rotation (Zhao et al., 2017; Ji et al., 2021b), chemical fumigation (Liu et al., 2022), and high-temperature steaming (Yang et al., 2019) have been documented to reduce the replanting problem.

Soil amendment is an environmentally benign alternative approach to chemical fumigation that is efficient in suppressing soil-borne diseases (Zhou et al., 2019; Lopes et al., 2022). Soil amendment involves soil incorporation with organic materials and covering it with a polyethylene film for at least two weeks at an optimal temperature (Momma et al., 2006). Soil amendment improves soil suppressiveness by reshaping the soil microbiome and soil physicochemical properties (Lopes et al., 2022), which ultimately results in the enrichment of beneficial microbes for plant disease control, direct suppression of bacterial and fungal pathogens (Zhou et al., 2019; Zhao et al., 2020), and modulation of the plant immune system (Jayaraman et al., 2021).

Soil amendment successfully mitigates replanting problems in apples, prunus, and strawberries (Browne et al., 2018; DuPont et al., 2021; Giovannini et al., 2021). However, the efficacy of soil amendment varies with the type of carbon source utilized for incorporation (Zhao et al., 2020) and is mediated by the microbiota, which impacts the emission of volatile compounds toxic to soil-borne pathogens (Poret-Peterson et al., 2019; Lopes et al., 2022). More importantly, cow dung, an organic amendment, is a cheap and easily available resource that improves plant and soil health, resulting in sustainable crop production (Gupta et al., 2016). Integrating soil amendment and biofumigants, such as *Brassica* spp., effectively control apple replanting problems (Wang and Mazzola, 2019). However, the effect of soil amendment with either cow dung or canola (biofumigant) on GRP and the taxonomic and functional diversity of the associated bacterial and fungal communities remains unclear. Thus, we aimed to determine the impact of cow dung, and canola on soil microbial communities and GRP reduction in six-year-old ginseng cultivated soil, which had a significant GRP.

## 2 Materials and methods

### 2.1 Materials, study design, and sampling

In this study, we obtained the soil from a six-year-old continuously cultivated ginseng farm in Punggi, Gyeongsangbuk-do Province, South Korea (36°48′37′′N,128°32′28′′E). The farm was abandoned because of the high GRP. Cow dung (pH = 8, electrical conductivity (EC) = 0.823) was obtained from a dairy farm in Daegu, Kyungpook-do Province, South Korea (32°32′27′′N,126°35′27′′E). Canola seed meal was acquired from the FarmHannong Bio Company in Seoul, Yeongdeungpo-gu, South Korea.

An 8-mm sieve was used to homogenize the collected soil thoroughly. Cow dung and canola seed powder were mixed separately with the soil at 1% in a plastic box (0.5 × 0.5 m in width and length). Non-amended soil served as the control. The treatments, including control, were watered at 70% field capacity and covered with plastic transparent polythene film for 40 days. The soil was left for a month at air temperature, and the polythene film was uncovered and air-dried in a greenhouse for one month. The greenhouse pots (31 cm height, 15 cm diameter) were filled with 2 kg of soil. Three one-year-old baby ginseng plants were transplanted into each plastic pot. All the soil amendments were arranged in a completely randomized design. The experiment was replicated three times, each containing five pots. Ginseng was grown for three months in a greenhouse. The seedlings were irrigated once a week.

**Figure 1:**
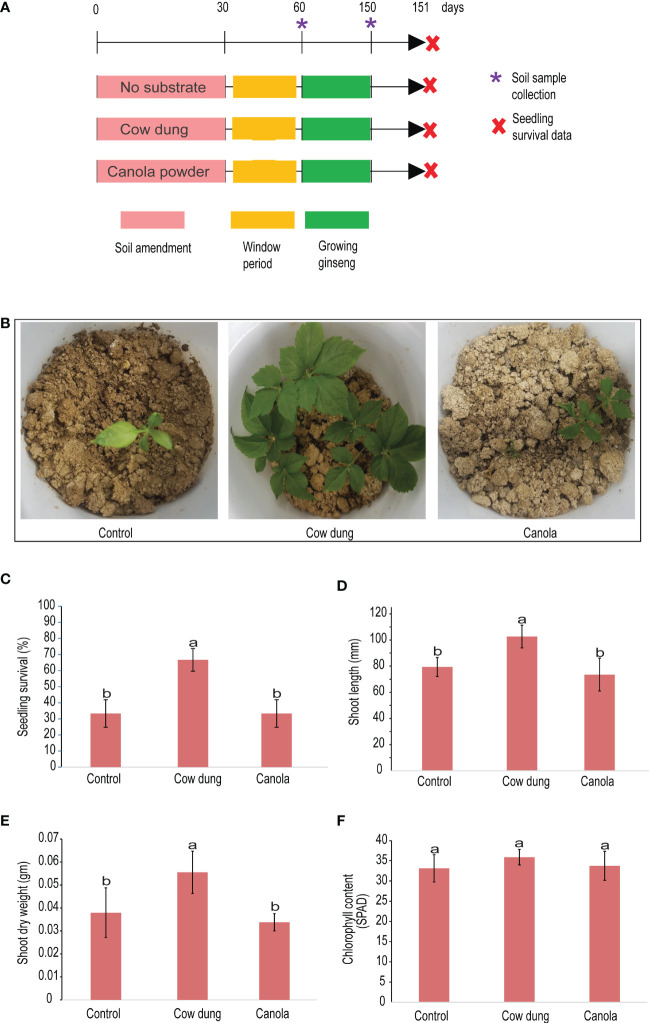
Impact of soil amendment on ginseng survival and plant growth properties. Pictorial view of the experimental scheme **(A)** and survived transplanted ginseng seedlings **(B)**. Graph showing the effect of different soil amendments on ginseng growth parameters during the three months after transplanting: the survival rate of transplanted ginseng seedlings **(C)**, shoot length **(D)**, shoot dry weight **(E)**, and chlorophyll content **(F)**. Mean values having the same letter(s) are not significantly different (p< 0.05). Error bars indicate standard deviation (n = 18).

Soil for DNA extraction was sampled by removing the top 2 cm of soil at a depth of approximately 5 cm from 7 cm away from each transplanted ginseng seedling at three points from each pot immediately before ginseng seedling transplanting (0 days after transplanting, 0DAT) and at the end of the experiment (90 days after seedling transplanting, 90DAT) (**Figure 1A**). The samples were pooled to obtain three samples (replicates) for each treatment and stored at −80°C until used for DNA extraction. For chemical property profiling, soil samples were collected at 0DAT.

The emergence rate was calculated as the number of emerging transplants divided by the total number of transplants. Ginseng replanting problem (GRP) was determined in each replication by dividing the number of surviving transplants by the total number of transplanted seedlings at 90DAT (Li et al., 2019). The GRP was expressed as percent seedling survival.

### 2.2 Soil chemical property analysis

The soil chemical properties were analyzed according to NIAST (2010) at Kyungpook National University, South Korea. A pH and EC meter (SP2000, Skalar BV, Netherlands) was used to measure the soil pH and EC. A titration method using an automatic titrator (Metrohm 888, Switzerland) was employed to assess the soil organic matter (SOM) content. Soil ammonium ( ${\text{NH}}_{4}^{+}$ ) and nitrate ( ${\text{NO}}_{3}^{-}$ ) concentrations were measured using cadmium reduction and salicylate colorimetric methods, respectively, using BLTEC QuAAtro (BLTEC KK, Osaka, Japan). The method employed by Dumas was used to determine the total nitrogen (TN) concentration with S832DR (Leco, USA). The concentrations of exchangeable potassium (K) and available P_2_O_5_ (AP) in the soil were measured using a PerkinElmer Optima 8300 ICP-OES (PerkinElmer, MA, USA) and SKALAR San++ system autoanalyzer (Skalar Analytical B.V., Breda, Netherlands), respectively. The soil cation exchange capacity (CEC) was measured using the BaCl_2_-H_2_SO_4_ exchange method.

### 2.3 Community-level physiological profiling

The change in the metabolic activity of the microbial communities in soil amendments was determined using the community-level physiological profiling (CLPP) method with Biolog EcoPlates with three replications. The EcoPlates had 31 kinds of carbon sources (Biolog, Hayward, CA, USA). Briefly, a one-gram soil was mixed with 99 mL of sterile distilled water. The mixture was vortex-homogenized for 20 min, and the soil particles were allowed to settle at 4°C for 30 min. The clay particles suspended in the supernatant were removed using one-gram CaCo_3_ and CaCl_2_. The supernatant (150 µL) was then dispensed into each well of a Biolog EcoPlate. The plates were then incubated at 25°C for 120 h under dark conditions. The color readings were measured at 590 nm and 750 nm wavelengths every 24 h using a Biolog microplate reader with Biolog MicroLog™ Software and MicroStation™. The absorbance value of each carbon source well was calculated by subtracting the value obtained from the control well at each time point. The carbon source utilization of the microbiota in each soil amendment was determined based on the percentage of total carbon utilization.

### 2.4 DNA extraction, library preparation, and sequencing

The DNeasy^®^ PowerSoil^®^ Pro Kit (Qiagen, Hilden, Germany) extracted microbial DNA from 0.5 g soil samples according to the manufacturer’s guidelines. DNA purity was checked with UV spectrophotometry (NanoDrop™ One^C^ spectrophotometer, Thermo Fisher Scientific) and double-checked using gel electrophoresis. DNA quantity was measured using a Qubit^®^ 2.0 Fluorometer (Life Technologies, Carlsbad, CA, USA). The DNA samples were kept at −80°C until used for library preparation for Illumina MiSeq sequencing.

The extracted DNA template was amplified using two-step PCR with universal primers 515F/907R for the bacterial 16S rRNA gene and ITS86F/ITS4R for the fungal internal transcribed spacer ITS (ITS1) region. Detailed descriptions of the primers and PCR conditions are provided in **Table S1**. The AMPure XP bead purification kit (Beckman Coulter, CA, USA) and Nextera^®^XT Index Kit (Illumina, San Diego, CA, USA) were used for the cleanup library and ligation processes, respectively. The pooled library was quality checking using an Agilent 2100 Bioanalyzer (Santa Clara, CA, USA). Sequencing was performed using the Illumina MiSeq platform (Illumina) at Kyungpook National University’s NGS Core Facility (Daegu, South Korea).

### 2.5 Bioinformatics analysis

The QIIME2 pipeline (https://qiime2.org) was used to demultiplex the raw sequences of bacteria and fungi from each sample. The reads were denoised using DADA2 in QIIME2 (Callahan et al., 2016) by removing singletons and chimeric sequences. Taxonomic assignment of the representative sequences, which were truncated and had high-quality scores, was performed using a classify-sklearn-based QIIME feature classifier trained on reference databases. SILVA (version 138.1) 99% full-length database (Quast et al., 2013) and UNITE database (version 8.3) (Nilsson et al., 2019) were used for bacteria and fungi, respectively. Taxonomy-assigned contaminants of chloroplasts, mitochondria, and kingdom-level unclassified taxa of ASVs (amplicon sequence variants) were excluded from downstream analysis. The sample reads were rarefied to an equal size (**Figure S1**) to a subsampling depth of the smallest 5551 and 20753 reads per sample of bacteria and fungi, respectively, to enable similarity comparison between treatments and avoid variation attributed to the DNA extraction method as well as library preparation. The normalized dataset contained 2102 and 232 ASVs.

The ecological functions of bacterial, fungal, and communities following soil amendment were determined using the functional annotation of prokaryotic taxa (FAPROTAX) (Louca et al., 2016), fungi functional guild (FUNGuild) (Nguyen et al., 2016), respectively.

### 2.6 Statistical data analysis

The R statistical software (version 4.1.3) performed all downstream statistical data analyses (R Core Team, 2022). Different R packages, ggplot (Wickham, 2016) and ComplexHeatmap (neatmap v2.1.0) (Gu et al., 2016), were used to visualize the data. Levene’s test (Oksanen et al., 2020) and PERMDISP (Anderson et al., 2011) were employed to determine the homogeneity of the variance and multivariate homogeneity of dispersion, respectively. The Shapiro–Wilk test was used to validate the data normality assumption. Comparison of the statistical difference between soil amendments in ginseng emergence, survival rate, and alpha diversity indices (at ASV level) was performed with ANOVA, and means comparison was performed with the least significant difference using the dplyr package. Factorial analysis was performed to additionally take into account the impact of the soil sampling time. The statistical differences among soil amendments in terms of bacterial and fungal community assembly were analyzed using permutational multivariate ANOVA (PERMANOVA) (Adonis; vegan, version 2.5.7) (Dixon, 2003). Differential abundance tools, such as ALDEx2 (Fernandes et al., 2013), LEfSe (Segata et al., 2011), metastat (White et al., 2009), metagenomeSeq (Paulson et al., 2013), and random forest in R (Beck and Foster, 2014) were used investigate potential microbial biomarkers in different soil amendments.

Random matrix theory (RMT) analysis based on the correlation method (Spearman’s rank correlation) from compositional data at the ASV level was employed to construct a network of bacterial and fungal communities. ASVs were rarefied, values less than 0.1% relative abundance were filtered out, and the default correlation coefficient cutoff point was set to 0.7 at p< 0.01. Gephi (version 0.9.2) software (Bastian et al., 2009) was used to visualize the co-occurrence network.

## 3 Results

### 3.1 Effects of soil amendments on soil nutritional content and ginseng survival rate

The impact of cow dung and canola on the soil nutritional content of six-year-old ginseng cultivated soil with a known problem with ginseng replanting is illustrated in **Table 1**. Although each treatment began with a single composite sick soil sample, the cow dung and canola soil amendments improved the soil’s nutritional status in terms of pH, EC, total carbon, total phosphorus, and available phosphorus. In addition, cow dung outperformed canola and control considerably (p ≤ 0.05) in terms of soil ${\text{NO}}_{3}^{-}$ , whereas canola exceeded in TN, ${\text{NH}}_{4}^{+}$ , and exchangeable potassium (K).

**Table 1:** Effects of soil amendment with cow dung and canola on soil chemical properties of ginseng soil with replanting problem^a^.

	Treatment		
	Control	Cow dung	Canola
pH	6.2 ± 0.00^c^	7.0 ± 0.06^a^	6.7 ± 0.06^b^
EC ^(^dS m^-1^)	0.30 ± 0.03^b^	0.60 ± 0.12^a^	0.80 ± 0.10^a^
CEC (cmol_c_ kg^-1^)	9.9 ± 0.16^b^	10.3 ± 0.17^b^	10.9 ± 0.07^a^
Total N (g kg^-1^)	0.10 ± 0.01^c^	0.10 ± 0.00^b^	0.20 ± 0.01^a^
Total C (g kg^-1^)	0.5 ± 0.01^c^	0.7 ± 0.01^b^	1.5 ± 0.01^a^
Total P (g kg^-1^)	279.9 ± 3.79^b^	360.6 ± 10.27^a^	377.5 ± 11.91^a^
AP (mg kg^-1^)	47.6 ± 3.2^c^	142 ± 7.6^a^	90.1 ± 1.1^b^
${\text{NO}}_{3}^{-}$ (mg kg^-1^)	7.9 ± 3.04^b^	22.1 ± 6.33^a^	0.5 ± 0.06^b^
${\text{NH}}_{4}^{+}$ (mg kg^-1^)	7.8 ± 1.05^b^	7 ± 0.43^b^	87.7 ± 13.04^a^
K (cmol_c_ kg^-1^)	0.2 ± 0.01^c^	0.2 ± 0.01^b^	0.5 ± 0.02^a^

Electrical conductivity (EC), cation exchange capacity (CEC), total nitrogen (Total N), total carbon (Total C), total phosphate (Total P), nitrate nitrogen ( ${\text{NO}}_{3}^{-}$ ), ammonium nitrogen ( ${\text{NH}}_{4}^{+}$ ), available P_2_O_5_ (AP), and exchangeable potassium (K).

Mean values followed by different letters (s) in a row represent significant differences at P ≤ 0.05, LSD test.

Our study also revealed that, between treatments, the ginseng survival rate, shoot length, and shoot dry weight varied significantly (p ≤ 0.05) (**Figures 1B–F**) at 90DAT. When compared to the control, cow dung significantly (p ≤ 0.05) increased ginseng survival by 100%, whereas canola showed no discernible difference (**Figures 1B, C**). Cow dung also had a significantly (p ≤ 0.05) high positive impact on ginseng seedling growth in terms of shoot length and weight (**Figures 1D, E**). The difference in chlorophyll content among the treatments was found to be insignificant (p > 0.05) (**Figure 1F**). Notably, there was no difference in ginseng emergence rate between treatments at the beginning; all had 100% emergence (data not shown), indicating that the soil amendments had no phytotoxic effects.

### 3.2 Microbial diversity and community composition changes following soil amendments

**Figure 2:**
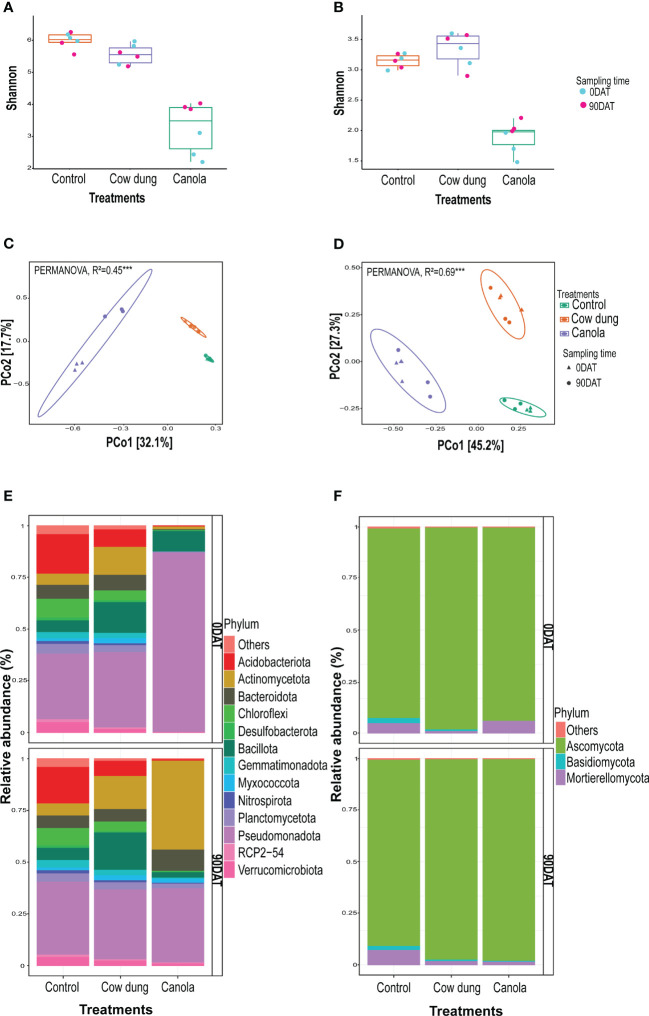
Impact of soil amendments on soil microbial diversity and community structure. Alpha bacterial **(A)** and fungal **(B)** diversities. Beta diversity of bacteria **(C)** and fungi **(D)** showing community structure of the treatments. Bacterial **(E)** and fungal **(F)** community composition (>1%) of treatments at phylum level on two sampling times.

Most alpha diversity indices revealed a significantly (*p* ≤ 0.05) high variation between the soil amendments, but not with sampling time (**Figures 2A, B**; **Table S2**). Furthermore, factorial ANOVA revealed that there was no significant (p>0.05) interaction effect between treatments and sampling time in most alpha diversity indices (**Table S2**), implying that the degree of soil amendment impact on these diversity indices did not vary across sampling times. Canola had a high negative effect on alpha diversity compared to cow dung and control (**Figures 2A, B**; **Table S2**). At 90DAT, there was a slight recovery of fungal and bacterial diversity in canola. Soil amendments also showed substantial variation in bacterial (R^2^ = 0.54, *p* ≤ 0.001) and fungal (R^2^ = 0.78, *p* ≤ 0.001) community composition (**Figures 2C, D**; **Table 2**). However, the shift in bacterial composition following cow dung and canola amendments across sampling periods varied, as revealed by the highly significant interaction effect between sampling time and treatment (R^2^ = 0.16, *p* ≤ 0.001) (**Table 2**). At 90DAT, the bacterial composition shift in cow dung was stable, whereas in canola, the microbial profile showed no return to that of 0DAT (**Figure 2C**, **Table 2**).

**Table 2:** PERMANOVA analysis of soil treatment effects on bacterial and fungal community composition structure based on weighted uniFrac distance.

Source of variation	df	16S		ITS	
		F. Model	R^2^	F. Model	R^2^
Soil amendment	2	8.62	0.45***	19.66	0.69***
Sampling time	1	2.83	0.07**	1.80	0.03
Soil amendment*Sampling time	2	2.98	0.16***	2.10	0.07
Residuals	12		0.32		0.21
Total	17		1.0		1.0

PERMANOVA: Permutational multivariate analysis of variance. Sampling time includes 0DAT (immediately before ginseng transplanting) and 90DAT (90 days after ginseng transplanting). df: degree of freedom. 16S: bacterial community based on the V4-V5 hypervariable region of the 16S rRNA gene. ITS: fungal community based on the internal transcribed spacer 1 (ITS1) region.

*p ≤0.05; **p ≤0.01; ***p ≤0.001.

Cow dung and canola had a remarkable impact on taxonomic composition at 0DAT and 90DAT, and they severely depleted members of the phylum Acidobacteriota, one of the dominant phyla in control (**Figures 2E, F**; **Table S3**). Following the canola amendment, Pseudomonadota, mainly Gammaproteobacteria, was the dominant population in the bacterial communities; however, Pseudomonadota was largely replaced by Actinomycetota at 90DAT (**Figure 2E**; **Table S3**). In addition, canola showed a severe transient negative impact on Bacteroidota abundance, although there was a complete recovery at 90DAT. In contrast, the cow dung amendment increased Bacillota and Actinomycetota. The increase in Bacillota abundance after cow dung could be partly attributed to the incorporation of cow dung as it was originally dominated by the Bacillota population (**Figure S2**). This effect persisted even after ginseng transplantation at 90DAT. Certain phyla, such as Armatimonadota and Desulfobacterota, vanished following canola treatment, and no members of this phylum were detected even at 90DAT. Myxococcota and Planctomcetota disappeared temporarily after canola treatment but later recovered three months later, at 90DAT. Ascomycota dominated the fungal community, with a relative abundance of over 85% across all treatments and sampling dates (**Figure 2F**). Among the other phyla, Basidiomycota was significantly negatively affected by the canola application. The second most abundant fungal phylum, Mortierellomycota, in control declined after cow dung and canola application at 90DAT. Ascomycota was the largest group of true fungi and comprised both pathogenic and saprophytic members. The fungal classes Leotiomycetes and Sordariomycetes dominated control. On both sampling dates, the relative abundance of Leotiomycetes decreased dramatically in cow dung (**Figure S3**).

### 3.3 Identification of potential microbial biomarkers enriched in soil amendments

**Figure 3:**
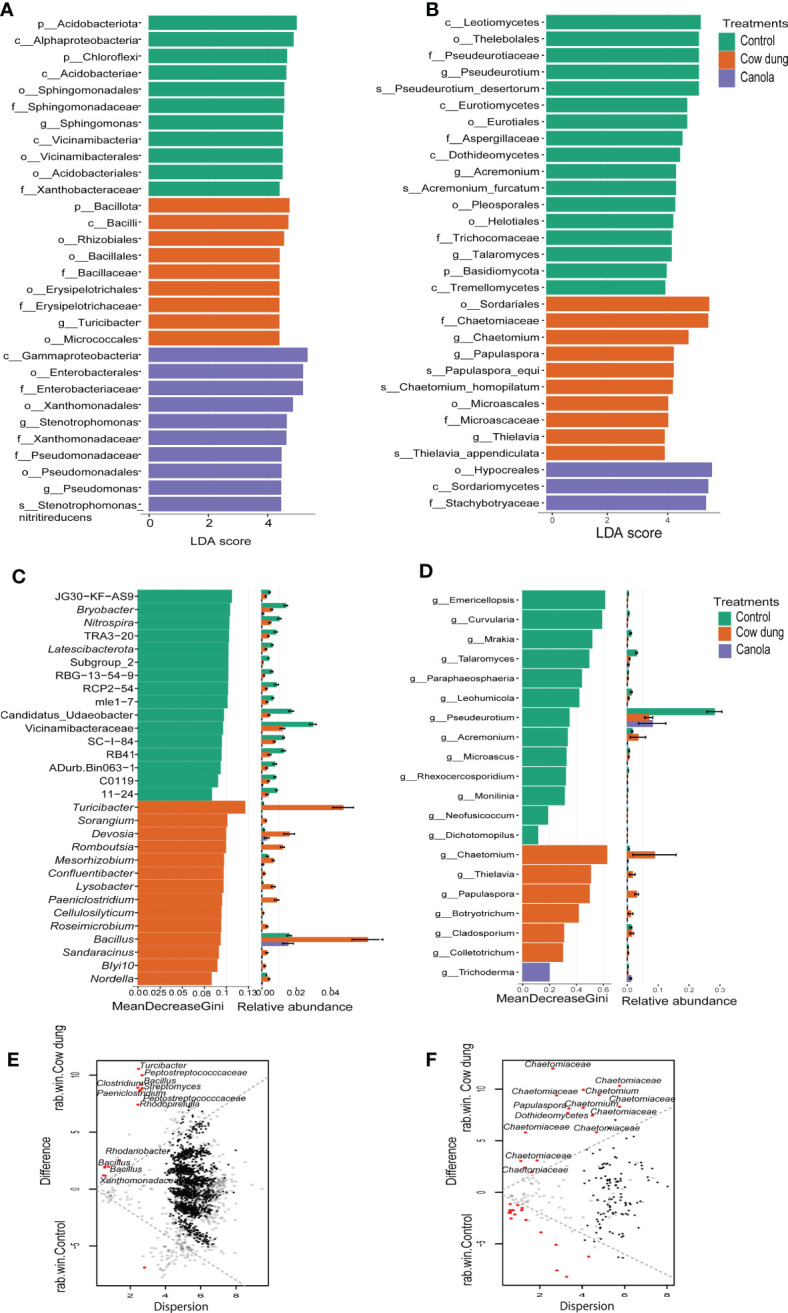
Differential abundance analysis of bacterial and fungal taxa after soil amendments. LEfSe analysis (LDA score > 4.0) displaying differentially abundant bacterial **(A)** and fungal **(B)** taxa among the treatments. Taxon names are abbreviated as p: phylum, c: class, o: order, f: family, and g: genus. Random forest model displaying the most predictive bacterial **(C)** and fungal **(D)** genera following soil amendments. Bland-Altman plot showing ALDEx2 analysis between cow dung and control of bacteria **(E)** and fungi **(F)**. ASVs with a larger difference and small dispersion are considered differentially abundant (q ≤ 0.1) and are indicated by red dots. Black and grey dots represent rare and abundant taxa, respectively.

ALDEx2, LEfSe analysis (LDA values > 4), the random forest model, metastat, and metagenomeSeq were used to identify potential microbial biomarkers discriminating the bacterial and fungal communities between the treatments. The results showed that over 464 bacterial and 75 fungal taxa were key discriminatory microbes between the treatment groups (**Figure 3**; **Supplementary material 2**). *Bacillus*, *Turicibacter, Streptomyces*, *Rhodanobacter*, and *Paeniclostridium* were the most highly stimulated bacterial genera in cow dung, as consistently detected by most biomarker selection tools (**Figures 3A, C, E**). Several features from Pseudomonadota, such as *Stenotrophomonas* and *Pseudomonas*, were the key bacteria found to be enriched in canola (**Figure 3A**). In control, the relevant members of p_Acidobacteriota and p_Chloroflexi were comparatively more abundant.

In the fungal community, most biomarker tools detected *Chaetomium* as the most significantly enriched genus in the cow dung application (**Figures 3B, D, F**), signifying that *Chaetomium* can be considered a key biomarker. Furthermore, f_ Stachybotryaceae, and *Trichoderma* were abundant in the canola treatment (**Figures 3B, D**). Members of c-Leotiomycetes (including *Pseudeurotium*), *Talaromyces*, *Mrakia*, and *Curvularia* were differentially abundant in control than in the substrate-amended soils (**Figures 3B, D**, **Supplementary material 2**).

### 3.4 Microbial co-occurrence network patterns and functional diversity alterations by soil amendments

**Figure 4:**
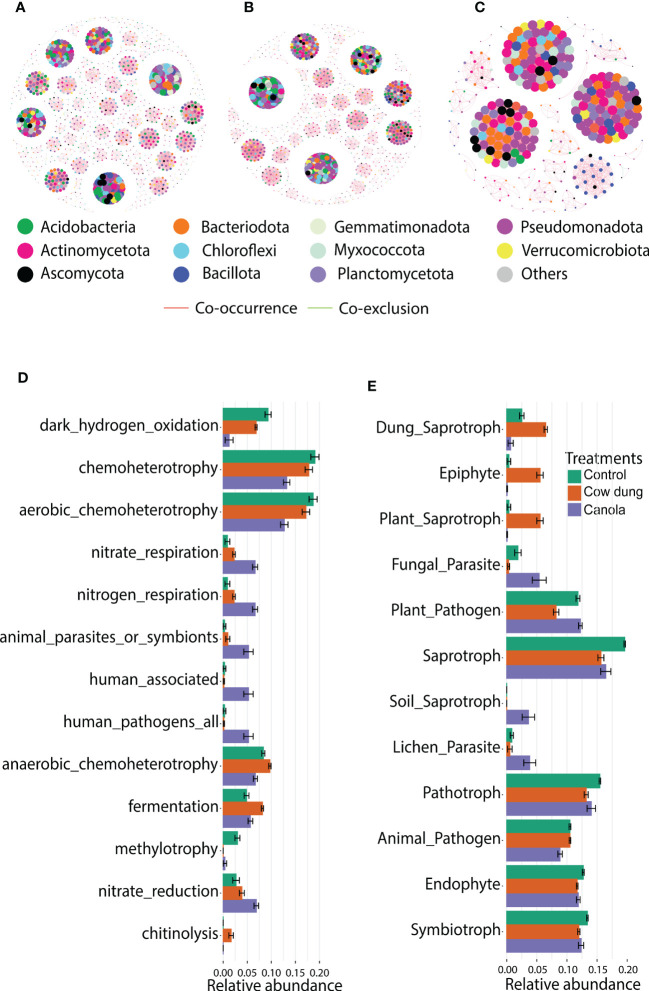
Impact of soil amendments on microbial co-occurrence network patterns and functional diversity. Co-occurrence networks of bacterial and fungal communities in treatments based on RMT analysis at the ASV level (Spearman’s rank correlation (corr_cut = 0.7), p< 0.05): control **(A)**, cow dung **(B)** and canola **(C)**. Node size in each treatment is proportional to the degree. Predicted functions of bacterial **(D)** and fungal communities **(E)** in different treatments based on the FAPROTAX database and fungal guild tools, respectively. LEfSe analysis (LDA score > 4.0) showed differentially enriched predicted functions across treatments.

RMT-based analysis was employed to construct co-correlation networks for the soil amendments. The results revealed that soil amendments showed a remarkable variation in bacterial-fungal network topologies (**Figures 4A−C** and **Table 3**). Cow dung shifted the microbial community assembly of the six-year-old ginseng soil to a highly connected (total links, avWD) and less modularized network (**Table 3**). It is worth noting that cow dung had a lower percentage of negative links, indicating better co-occurrence than the co-exclusion of bacteria and fungi. In contrast, in canola, the number of nodes, total links, avWD, modularity, GD, and modules were lower than in cow dung and control, implying that the impact of soil amendments on the complexity of the microbial co-occurrence network highly depends on the type of amendment. A negative relationship was found in the intra- and inter-phylum (**Figures 4A–C**).

**Table 3:** Co-occurrence network topological properties of bacterial-fungal communities in soil amendments.

	Soil amendment		
	Control	Cow dung	Canola
Total nodes	1303	1203	307
Total links^a^	20568	35544	5167
Total positive links (%)	99.9	99.97	99.94
Total negative links (%)	0.1	0.03	0.06
avWD^b^	31.57	59.09	33.66
Graph density (GD)	0.024	0.049	0.11
Modularity	0.875	0.784	0.723
Modules	172	124	31

a Links: pairwise correlation of nodes.

b Average weighted degree (avWD): average number of links per treatment.

Given the shift in soil microbial communities after soil amendment with cow dung and canola, FAPROTAX analysis was performed to determine functional changes. Chemoheterotrophy and aerobic chemoheterotrophy putative functions were the most dominant in all treatments (**Figure 4D**). The canola amendment enhanced functions related to nitrate reduction and nitrate/nitrogen respiration, which are often called dissimilatory nitrate reduction and lead to the accumulation of ${\text{NH}}_{4}^{+}$ (Picazo et al., 2021) (**Figure 4D**). There was also a remarkable reduction in the functions related to animal parasites and human-associated and human pathogens following canola. Furthermore, fermentation and chitinolysis were enhanced in cow dung (**Figure 4D**). The change in fungal ecological guild following soil amendments was parsed using FUNGuild (**Figure 4E**). The functional profiles of ginseng soil changed with the soil amendments based on the trophic mode. Cow dung-amended soil and cow dung were enriched with dung saprotrophs, epiphytes, and plant saprophytes. In addition, the same treatment was less enriched with functions related to plant pathogens and fungal parasites than canola and control, indicating its potential to enhance soil suppressiveness. The genera that contributed to plant pathogens were *Acremonium*, *Alternaria*, *Calonectria*, *Colletotrichum*, *Coniochaeta*, *Cladosporium, Curvularia*, *Dendryphion*, *Fusarium*, *Microascus*, *Monilinia*, *Neofusicoccum*, *Neonectria*, *Rhexocercosporidium*, *Stagonospora*, and *Trichoderma*.

### 3.5 CLPP analysis and the soil microbiota-soil nutrient relationship

**Figure 5:**
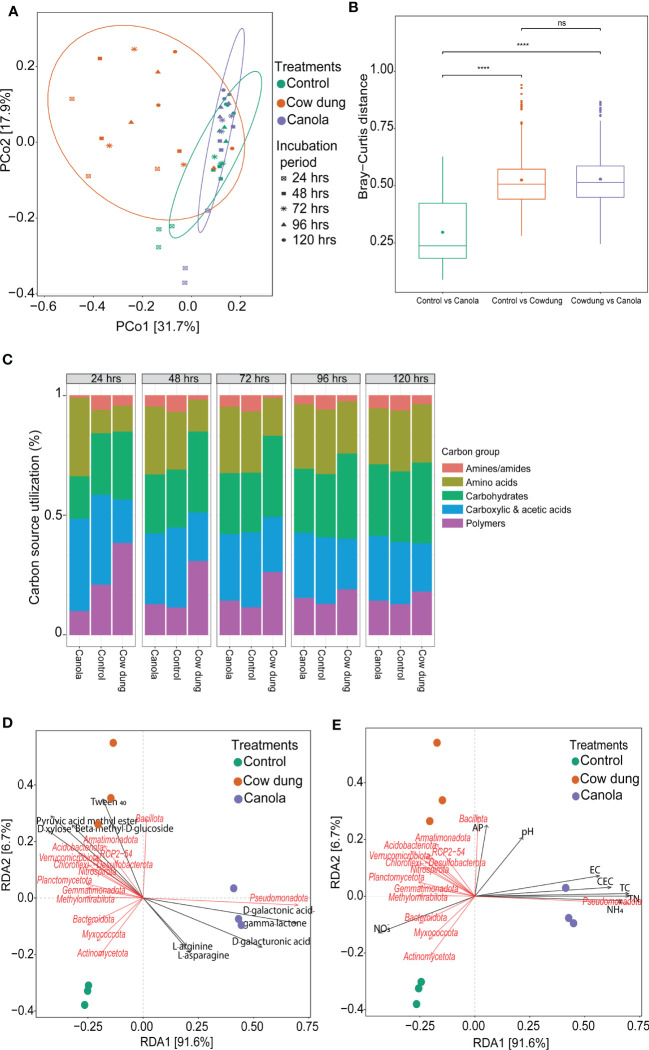
Effects of soil amendments on soil chemical properties, microbial communities, and their metabolic activities. PCOA plot showing the community-level physiological profiling using Biolog EcoPlates with 31 different carbon sources at different incubation periods **(A, B)**. Relative carbon source utilization (%) **(C)**. Distance-based redundancy analysis (dbRDA) based on Bray-Curtis distance displaying the relationship of the soil bacterial communities with metabolic activities **(D)** and soil chemical properties **(E)** as indicated by the angles and projection of arrows. The length of the arrows represents the contribution of each soil chemical parameter to the variation in the bacterial community structure. Refer to **Table 1** for soil chemical property abbreviations.

CLPP analysis using 31 different carbon sources in Biolog EcoPlates showed that cow dung induced significant functional changes related to carbon utilization (**Figures 5A, B**). The distribution of carbon source utilization in the soil amendments differed (**Figure 5C**). Amines/amides were the least utilized carbon groups by the soil microbial communities in the soil amendments. More importantly, compared to control and canola, the cow dung-amended soil had higher utilization rates of carbohydrates and polymer carbon sources. Among the polymers and carbohydrates, Tween 40 and beta-methyl-D-glucoside were the most utilized carbon sources in cow dung (**Figure S4**). These carbon sources were highly associated with Bacillota (**Figure 5D**). In contrast, cow dung showed less catabolic activity of carboxylic and acetic acids than canola. Canola showed higher metabolic activities of D-galactonic acid and D-galacturonic acid, which were positively associated with Pseudomonadota (**Figure 5D**). Such differences in metabolic activity following the change in microbial community assembly may be linked to modifications in soil nutritional content after soil amendment. Most soil chemical properties, including TN, total carbon, and CEC, strongly influenced the soil microbial community structure, where improved soil pH and AP had a cow dung-mediated positive influence on Bacillota (**Figure 5E**; **Table S4**). Similarly, most soil factors were consistently found to be determinant factors in the fungal community assembly (**Table S4**).

## 4 Discussion

The GRP is a major concern for ginseng farmers throughout much of the world, including South Korea. Soil microbiota imbalance and accumulation of toxic substances are the major causes of GRP (Lee et al., 2015; Liu et al., 2021b). Several agricultural interventions have the potential to shift the microbiota structure and induce disease suppression (Qi et al., 2020) (Jayaraman et al., 2021). soil amendment is an environmentally benign non-chemical approach that is effective in reshaping the soil microbiota, resulting in reduced continuous cropping obstacles (Lopes et al., 2022) owing to the introduction or activation of beneficial microbes for soil-borne disease control (Zhao et al., 2020). However, the impact of cow dung and canola on GRP has not been previously investigated, and the shift in taxonomic and functional diversity of soil bacterial and fungal communities after cow dung remains unclear. Therefore, the present study investigated the effect of cow dung and canola on GRP reduction, soil nutrition, and microbiota.

The survival rate of transplanted ginseng seedlings grown in cow dung-treated soil was 100% higher than that of control, whereas canola had no significant effect. In support of our results, numerous studies have documented that soil amendment alleviates the replanting problems of different plants (Browne et al., 2018). Li et al. (2019) also applied soil disinfestation with bean dregs and sugarcane bagasse and found a reduction in the replant failure of ginseng plants. However, we believe that this study is the first to document the alleviation of GRP using a soil amendment with cow dung as a substrate. Changes in soil microbiota are one of the major contributing factors affecting the occurrence of GRP (Dong et al., 2018). Thus, understanding and manipulating the soil microbiota with pre-planting agronomic practices is a novel approach to microbiome-assisted strategies to enhance soil suppressiveness for suitable organic farming systems (Dong et al., 2018; De Corato, 2020).The direct exogenous microbial input from the incorporated cow dung may have contributed to the alteration in microbial community structure assembly (Sun et al., 2015). More importantly, the enhanced soil nutrient content after incorporating cow dung as a carbon source could provide diverse microhabitats for soil microbial growth and colonization (Suleiman et al., 2016; Zhou et al., 2019; Ye et al., 2021), inducing substantial changes in the soil bacterial and fungal community structures (Hu et al., 2018; Liu et al., 2021b). Our findings are in agreement with previous reports that soil nutrients, such as pH, TC, and TN, were the major factors shaping soil microbial community in the ginseng field and alleviating GRP (Zhang et al., 2020; Ji et al., 2021b; Liu et al., 2021b).

Chloroflexi and Acidobacteriota are oligotrophic bacteria that specifically adapt to low available resources (Eo and Park, 2016; Liang et al., 2018; Ye et al., 2021), and low soil pH often decreases their abundance after organic amendments (Ye et al., 2021), similar to the cow dung and canola results. The accumulation of different types of acidic compounds after ginseng monocropping is a major challenge in ginseng production (Li et al., 2018). The improvement in pH after cow dung shows its neutralizing potential, as the applied cow dung was alkaline. Similarly, a previous study reported the acid-neutralizing potential of manure and its potential to prevent nitrate leaching (Sun et al., 2015). Chloroflexi is often highly correlated with several soil-borne diseases (Liang et al., 2018; Ren et al., 2018) and is described as disease-inducing bacteria (Niu et al., 2016). This may be due to the fact that the majority of Chloroflexi members are not able to fix nitrogen but instead compete with beneficial soil microbes and host plants for nitrogen resources (Niu et al., 2016; Ren et al., 2018). In contrast, members of Bacillota, including *Bacillus* and *Clostridium* were differentially abundant in cow dung treatment. Bacillota, known for their crop growth promotion and high antifungal activity, are often strongly associated with soil suppressiveness (Lee et al., 2021) and subsequently protect ginseng plant health. Microbial biomarkers, such as *Bacillus* and *Streptomyces*, have an inverse relationship with GRP, and they are significantly enriched in healthy ginseng soil (Li et al., 2019; Ji et al., 2021a). This was partly due to their ability to hydrolyze ginsenosides, one of the main components of GRP, *via* β-glucosidase and β-glucuronidase (Li et al., 2019). *Clostridium* and *Rhodanobacter* often increase with substrate soil amendment and improve soil suppressiveness (Liang et al., 2018; Poret-Peterson et al., 2019) *via* their toxic organic acids (Huang et al., 2015). In addition, *Rhodanobacter* degrades diisobutyl phthalate, a toxic allelopathic chemical that causes GRP (Dong et al., 2018). This suggests that the enrichment of such biomarkers in cow dung may have reduced obstacles to ginseng replanting (Ji et al., 2021a). In the fungal community, Leotiomycetes, which include diverse groups of plant pathogens and are often enriched in continuous ginseng cultivation (Bao et al., 2020), were found to be remarkably reduced by cow dung compared to control and canola. However, *Chaetomium*, a biomarker in healthy ginseng soil (Dong et al., 2022), was abundant in cow dung. *Chaetomium* is a beneficial fungus for controlling soil-borne diseases, including ginseng diseases (Grunewaldt-Stöcker and von Alten, 2003; Zhou et al., 2019; Li et al., 2020), and it also increases crop tolerance to abiotic stresses (Liu et al., 2021b). In canola treatment, the decline in the abundance of Gemmatimonadota, Armatimonadota, Desulfobacterota, Myxococcota, and Planctometota could be attributed to their sensitivity to ITCs released from soil biofumigants (Tagele et al., 2021).

Co-occurrence network analysis is a novel method that helps us to understand the complex association among microbial communities in soil ecosystems and highlights how such complex interaction is impacted by agricultural activities (Goberna and Verdú, 2022). The more clustered network and firmly connected microbial community in cow dung could potentially make the community highly stable (Sun et al., 2015) to soil sickness owing to continuous ginseng monocropping (He et al., 2021), pathogen colonization (Rybakova et al., 2017), and environmental stresses (He et al., 2021), thereby maintaining soil health (Liu et al., 2021a). In contrast, scattered niches in control and canola would harm the prompt defense against foreign pathogen invasion (Tao et al., 2018). Similar to our results, previous reports have documented larger modularity and a higher percentage of negative links in disease-conducive soil than in disease-suppressive soils, in which the latter has dense, high degree, and low modularity (Gao et al., 2021; Ji et al., 2021b). Furthermore, structural shifts in the microbiota are mostly associated with functional changes that drive the agricultural ecosystem (Dubey et al., 2015). Alterations in soil carbon pools can affect the metabolic activity of the soil bacterial and fungal communities. The high utilization of carbohydrate and polymer carbon sources and the predicted function of high fermentation, as seen in cow dung, increase the conversion of carbohydrates to organic acids (Yu et al., 2021), which can potentially reduce soil-borne pathogens, including those causing GRP (Poret-Peterson et al., 2019), although this requires further investigation. Furthermore, such high microbial metabolic activity coupled with plastic cover during BSD process can potentially enhance the soil temperature that may, in turn, contribute to a high decomposition rate of residual antibiotics (Cycon et al., 2019). The saprotroph trophic mode was the most dominant functional group in control, which agrees with previous reports that continuous ginseng cultivation leads to significantly enriched saprotrophs (Bao et al., 2020; Ji et al., 2021b). In addition, the reduced enrichment of predicted functions related to plant pathogens and fungal parasites in cow dung suggests that its application may have resulted in the enrichment of various ecological functions that contribute to reducing GRP.

## 5 Conclusions

Amending soil with cow dung improved soil nutritional content, increased ginseng survival, and showed a remarkable positive effect on ginseng shoot length and shoot. Microbial profiling by sequencing showed that cow dung modified the soil microbial composition, in which Acidobacteriota and Leotiomycetes were highly depleted, whereas Bacillota and Actinomycetota were enriched. Further microbial biomarker analysis revealed that *Bacillus*, *Turicibacter, Streptomyces*, *Rhodanobacter*, *Paeniclostridium*, and *Chaetomium* were the most highly stimulated genera in cow dung. The microbial communities in the cow dung soil had higher utilization rates of carbohydrates and polymer carbon sources, which were most highly associated with Bacillota. In contrast, in Pseudomonadota-dominated canola soil, there was a higher metabolic activity of carboxylic and acetic acids, mainly D-galactonic acid and D-galacturonic acid. After soil substrate amendment, soil nutritional changes, such as soil TN, total carbon, and CEC, were found to be determinant factors in microbial community composition. Furthermore, RMT analysis revealed that cow dung transformed the microbial co-occurrence network into a highly connected and complex network. In contrast, canola harmed the complexity of the microbial co-occurrence network. The predicted functional profiles of ginseng soil, based on FUNGuild trophic mode, showed that cow dung was less enriched with functions related to plant pathogens and fungal parasites compared to canola and control, signifying its potential to enhance soil suppressiveness.

## Data availability statement

The original contributions presented in the study are publicly available. This data can be found here: NCBI, PRJNA846516 (BioSample accessions: SRR19568260-SRR19568295, SRR21733898–SRR21733899).

## Author contributions

ST and J-HS planned and designed the research study. ST, R-HK, MJ, D-RJ, KL, DL, and WK performed the research. ST and J-HS analyzed the data. ST prepared the figures and tables. ST and J-HS wrote the manuscript. All authors contributed to the article and approved the submitted version.

## Supplementary material

Supplementary material 2 Bacterial and fungal biomarkers detected using Aldex2, metastat, and metagenomeSeq.
