# Supplementary material for: Soil amendment with cow dung modifies the soil nutrition and microbiota to reduce the ginseng replanting problem
Source: Front Plant Sci. 2023 Jan 24;14:1072216. doi: 10.3389/fpls.2023.1072216 (PMC9902886; doi:10.3389/fpls.2023.1072216)
Supplement: Supplementary file 1 [file DataSheet_1.docx]

Supplementary Material

Soil Amendment with Cow Dung Modifies the Soil Nutrition and Microbiota to Reduce the Ginseng Replanting Problem

**Setu Bazie Tagele^1, 3^, Ryeong-Hui Kim^2^, Minsoo Jeong^1^, Kyeongmo Lim^1^, Da-Ryung Jung^1^, Dokyung Lee^2^, Wanro Kim^1^, and Jae-Ho Shin^1, 2, 3*^**

^1^Department of Applied Biosciences, Kyungpook National University, Daegu 41566, Republic of Korea

^2^Department of Integrative Biology, Kyungpook National University, Daegu 41566, Republic of Korea

^3^NGS core facility, Kyungpook National University, Daegu 41566, Republic of Korea

***Correspondence:** Jae-Ho Shin

[jhshin@knu.ac.kr](mailto:jhshin@knu.ac.kr)


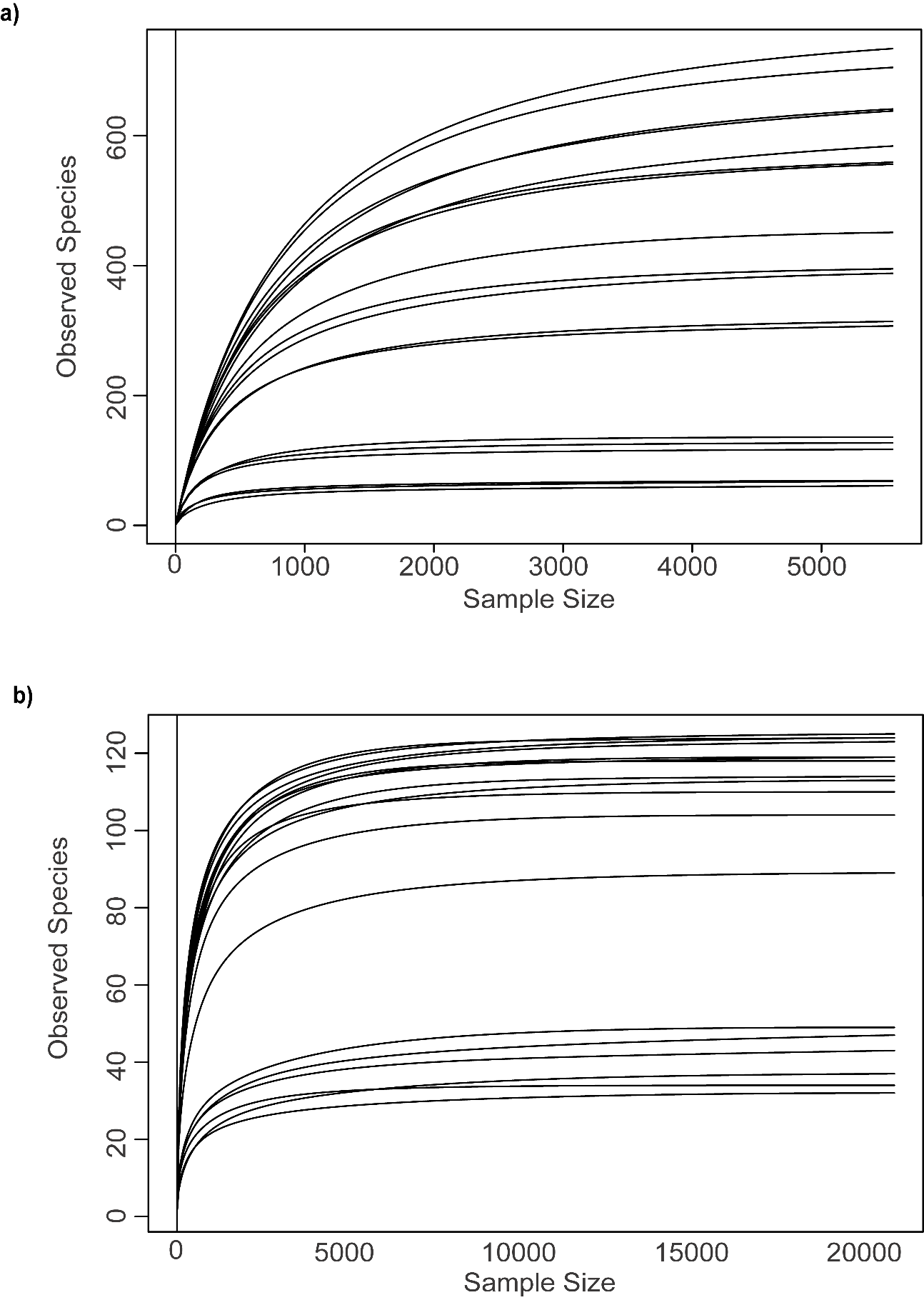


**Figure S1** Rarefaction curve of bacterial (a), and fungal (b) observed species in 18 soil samples of the three BSD treatments at two sampling times.


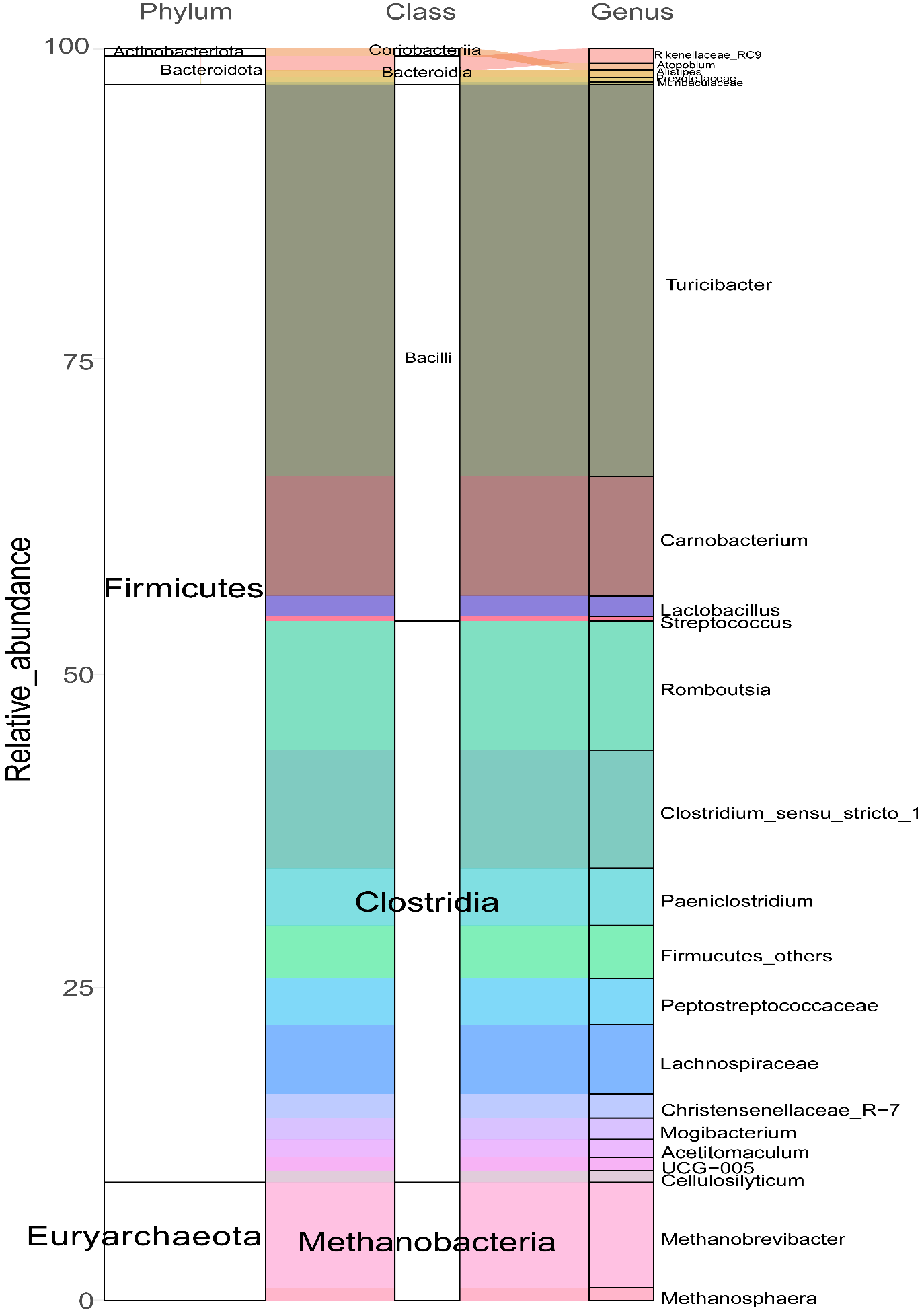


**Figure S2** Bacterial community composition of fresh cow dung used in the BSD−CD treatment.


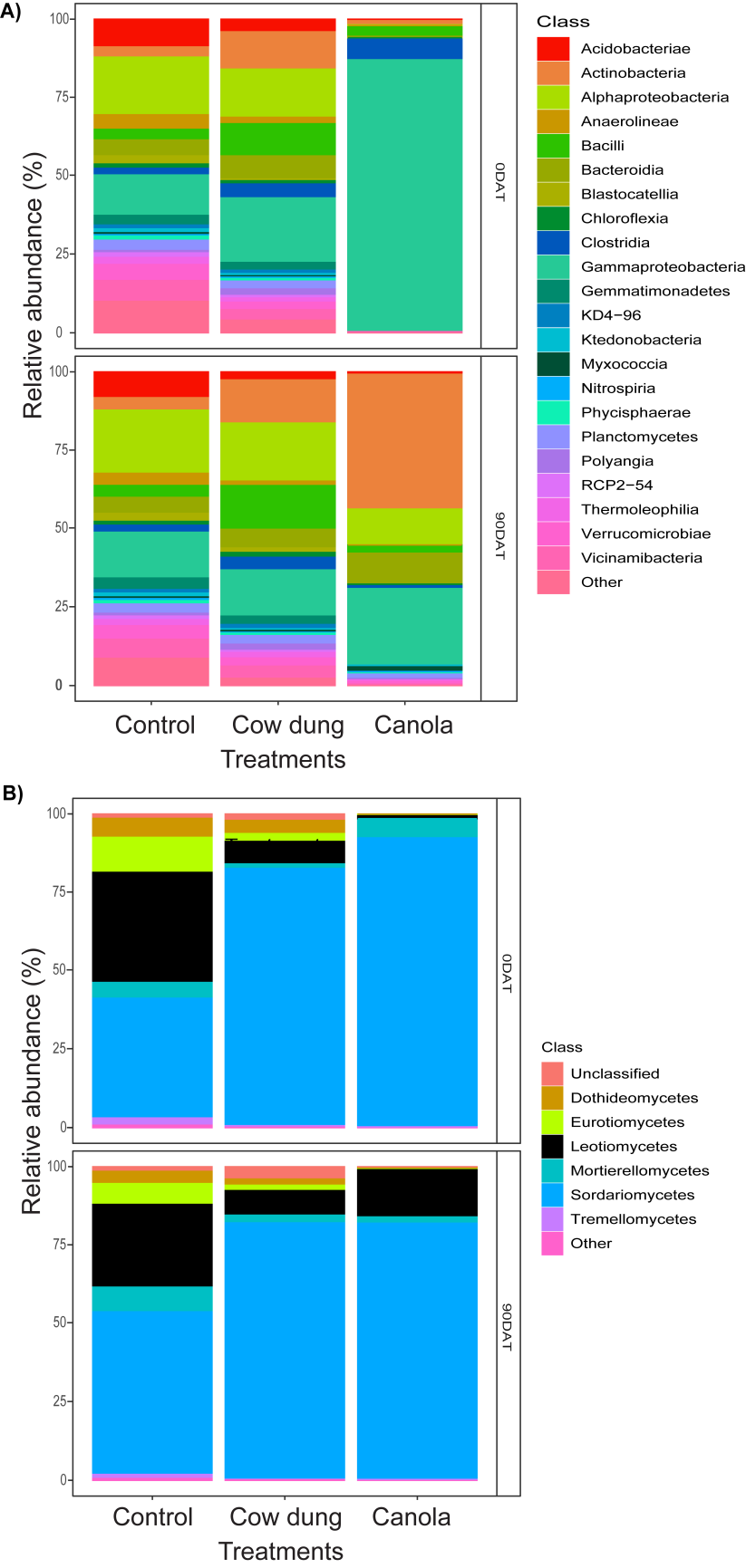


**Figure S3** Relative abundance of bacterial and fungal communities at the class level in BSD treatments.

##
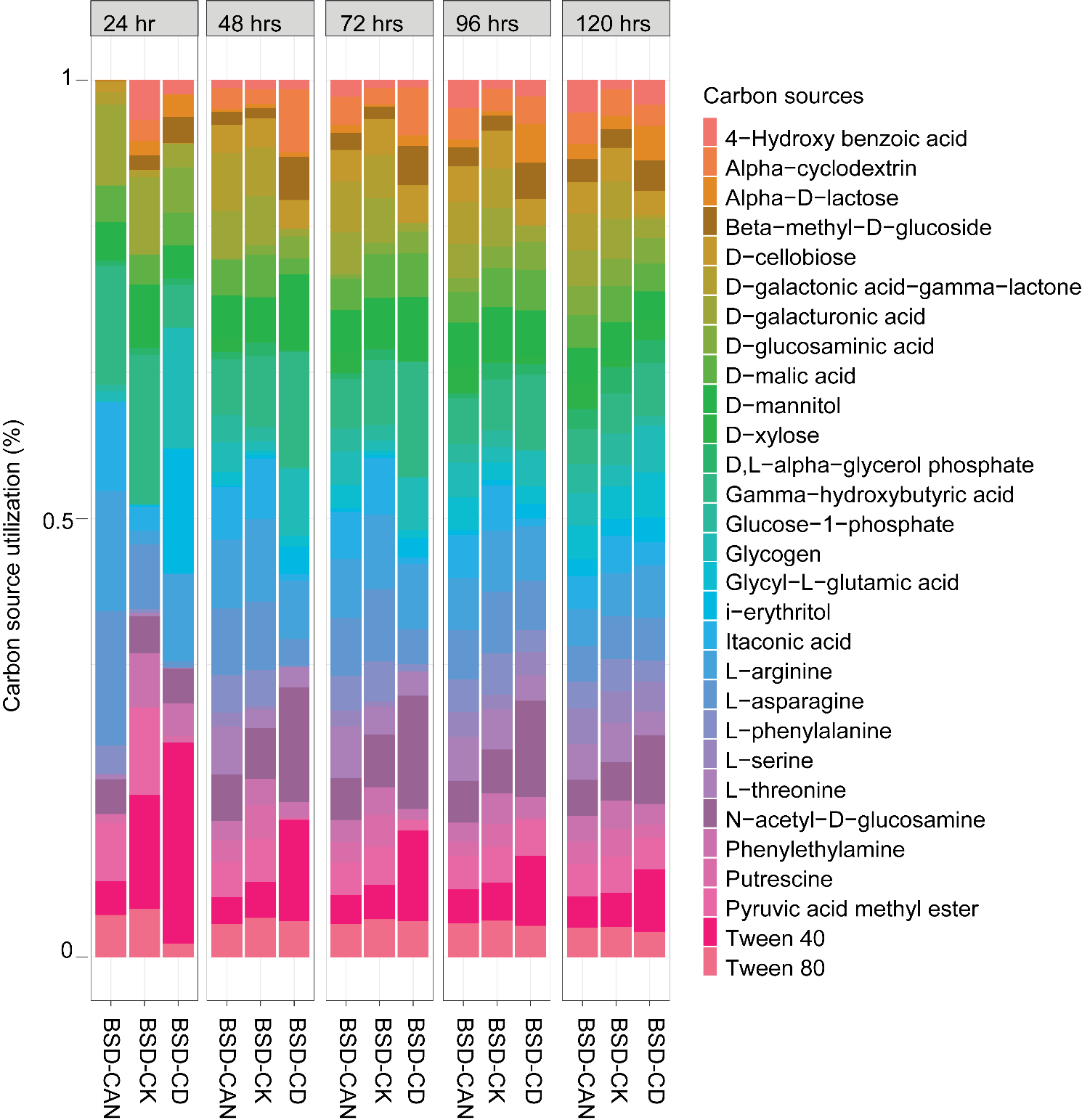


**Figure S4** Community-level physiological profiling using Biolog EcoPlates with 31 different carbon sources at different incubation periods.

.

**Table S1** Sets of primers and PCR reaction conditions used in Illumina sequencing.

| **First round PCR** |  |  |  |
| --- | --- | --- | --- |
| Target gene | Primer set | Sequences (5’-3’) | PCR reaction conditions |
| **Bacteria** |  |  |  |
| V4-V5 region of the 16S rRNA gene | 515F | GTGCCAGCMGCCGCGGTAA | Initial denaturation at 95℃ for 3; followed by 20 cycles at 95℃for 30 s, at 56℃for 30 s, at 72℃for 30 s; final elongation at 72℃ for 5 min. |
|  | 907R | CCGYCAATTCMTTTRAGTTT |  |
| **Fungi** |  |  |  |
| ITS region | ITS86F | GTGAATCATCGAATCTTTGAA | Initial denaturation at 95℃ for 5; followed by 30 cycles at 95℃for 30 s, at 58℃for 30 s, at 72℃for 30 s; final elongation at 72℃ for 5 min. |
|  | ITS4R | TCCTCCGCTTATTGATATGC |  |
| **Second round PCR** |  | PCR reaction conditions |  |
| **Bacteria** |  | Initial denaturation at 95℃ for 5; followed by 10 cycles at 95℃for 30 s, at 57℃for 30 s, at 72℃for 30 s; final elongation at 72℃ for 5 min. | |
| **Fungi** |  | Initial denaturation at 95℃ for 5; followed by 10 cycles at 95℃for 30 s, at 57℃for 30 s, at 72℃for 30 s; final elongation at 72℃ for 5 min. | |
| **PCR reaction mixture for the two PCR** | | | |
| 25 µl EmeraldAmp MAX HS PCR Master Mix (Takara Bio, Shiga, Japan), 1 µl DNA template, 1 µl (0.5 µM/µl) of each primer, and 22 µl of ddH_2_O | | | |

**Table S2** Two-way factorial ANOVA analysis of BSD treatment, sampling time, and alpha diversity indices of bacterial and fungal diversity.

|  |  | **Sampling time** | | | | | | | |  | **p-value** |  |
| --- | --- | --- | --- | --- | --- | --- | --- | --- | --- | --- | --- | --- |
|  |  | **0DAT** | | |  |  | **90DAT** |  |  |  |  |  |
|  |  | **BSD−CK** | **BSD−CD** | **BSD−CAN** |  | **BSD−CK** | **BSD−CD** | **BSD−CAN** |  | **Treatments** | **Sampling time** | **Treatments * Sampling time** |
| **Bacteria** |  |  |  |  |  |  |  |  |  |  |  |  |
| Observed |  | 631±39.9 | 512±102.2 | 63.7±2.8 |  | 559.3±100.8 | 382.3±42.2 | 125±5.1 |  | <0.001 | 0.4055 | 0.3523 |
| Chao1 |  | 665±42.6 | 537.8±110.9 | 67.2±4.6 |  | 578.5±110.3 | 395.1±44.7 | 126.3±6.3 |  | <0.001 | 0.4142 | 0.3775 |
| ACE |  | 656.4±44 | 537.5±111.5 | 65.7±3.4 |  | 579.8±110.7 | 392.8±44.7 | 125.8±5.5 |  | <0.001 | 0.4062 | 0.3704 |
| Shannon |  | 6.1±0.1 | 5.6±0.2 | 2.5±0.3 |  | 5.9±0.2 | 5.4±0.1 | 3.9±0.1 |  | <0.001 | 0.0433 | 0.0014 |
| Simpson |  | 1±0 | 1±0 | 0.8±0.1 |  | 1±0 | 1±0 | 1±0 |  | 0.003 | 0.0.031 | 0.0156 |
| InvSimpso |  | 319.1±19.1 | 159.4±28.4 | 7.4±3.1 |  | 267.4±53.5 | 150±14.1 | 22.6±3 |  | <0.001 | 0.5592 | 0.4748 |
| Fisher |  | 191.7±17 | 146.8±37.5 | 10.3±0.5 |  | 165.2±40.5 | 97±14.1 | 23.3±1.2 |  | <0.001 | 0.3277 | 0.4737 |
| Coverage |  | 1±0 | 1±0 | 1±0 |  | 1±0 | 1±0 | 1±0 |  | 0.002 | 0.3910 | 0.3775 |
| PD |  | 156.1±4.7 | 132.9±17.3 | 17.6±1.8 |  | 146.2±18.9 | 107.2±9.8 | 40.7±2 |  | <0.001 | 0.7171 | 0.1655 |
| **Fungi** |  |  |  |  |  |  |  |  |  |  |  |  |
| Observed |  | 117.7±4.1 | 114.3±6.1 | 34.3±1.5 |  | 120.3±1.9 | 108.7±10.2 | 46.3±1.8 |  | <0.001 | 0.4977 | 0.2808 |
| Chao1 |  | 117.8±4.2 | 114.3±6.1 | 34.3±1.5 |  | 120.4±2 | 109.2±10.4 | 47.5±1.5 |  | <0.001 | 0.4305 | 0.2601 |
| ACE |  | 117.8±4.2 | 114.4±6.1 | 34.4±1.6 |  | 120.5±2.1 | 109.1±10.3 | 47.5±1.7 |  | <0.001 | 0.4341 | 0.2611 |
| Shannon |  | 3.1±0.1 | 3.4±0.2 | 1.7±0.2 |  | 3.1±0.1 | 3.3±0.3 | 2.1±0.1 |  | <0.001 | 0.3320 | 0.2786 |
| Simpson |  | 0.9±0.1 | 0.9±0.1 | 0.7±0.1 |  | 0.9±0.1 | 0.9±0.1 | 0.8±0.1 |  | <0.001 | 0.0354 | 0.1046 |
| InvSimpson |  | 8.3±1 | 13.4±1.6 | 3.8±0.5 |  | 9.2±0.3 | 15.6±3.3 | 5.5±0.5 |  | <0.001 | 0.2297 | 0.92576 |
| Fisher |  | 16.5±0.7 | 16±1 | 4±0.2 |  | 16.9±0.4 | 15.1±1.7 | 5.6±0.3 |  | <0.001 | 0.5813 | 0.3550 |
| Coverage |  | 1±0.1 | 1±0.1 | 1±0.1 |  | 1±0.1 | 1±0.1 | 1±0.1 |  | 0.6514 | 0.0783 | 0.3842 |
| PD |  | 14.4±0.3 | 12.5±1 | 5.4±0.2 |  | 14±0.2 | 12.8±1.1 | 7.4±0.4 |  | <0.001 | 0.1978 | 0.1865 |

**Table S3** Two-way factorial ANOVA analysis of BSD treatment, sampling time, and bacterial and fungal community composition at the phylum level.

|  | **Sampling time** | | | | | | | |  | **p-value** |  |
| --- | --- | --- | --- | --- | --- | --- | --- | --- | --- | --- | --- |
|  | **0DAT** | | |  |  | **90DAT** |  |  |  |  |  |
|  | **BSD−CK** | **BSD−CD** | **BSD−CAN** |  | **BSD−CK** | **BSD−CD** | **BSD−CAN** |  | **Treatments** | **Sampling time** | **Treatments * Sampling time** |
| **Bacteria** |  |  |  |  |  |  |  |  |  |  |  |
| Pseudomonadota | 32.2 ± 0.3 | 36.9 ± 1.19 | 87.4 ± 0.93 |  | 35.7 ± 3.74 | 34.2 ± 0.32 | 36.4 ± 4.1 |  | <0.01 | <0.05 | <0.05 |
| Bacillota | 6.2 ± 0.3 | 15.4 ± 2.85 | 10.4 ± 1.98 |  | 6.4 ± 0.38 | 18.6 ± 0.98 | 3.1 ± 0.49 |  | <0.001 | >0.05 | <0.05 |
| Bacteroidota | 6.4 ± 0.7 | 7.5 ± 2.04 | 0.5 ± 0.4 |  | 6.1 ± 0.94 | 6.1 ± 1.23 | 10.3 ± 2.29 |  | >0.05 | >0.05 | >0.05 |
| Chloroflexi | 9.5 ± 0.4 | 4.8 ± 1.04 | 0.1 ± 0.02 |  | 8.4 ± 0.62 | 4.8 ± 0.08 | 0.2 ± 0.15 |  | <0.001 | >0.05 | >0.05 |
| Acidobacteriota | 19.6 ± 0.8 | 8.5 ± 1.01 | 0.1 ± 0.04 |  | 18.2 ± 1 | 7.8 ± 0.76 | 0.4 ± 0.09 |  | <0.001 | >0.05 | >0.05 |
| Planctomycetota | 4.7 ± 0.04 | 3.4 ± 0.45 | 0 ± 0 |  | 4.1 ± 0.71 | 3.6 ± 0.43 | 2.3 ± 0.48 |  | <0.001 | <0.05 | >0.05 |
| Verrucomicrobiota | 5.3 ± 0.4 | 2 ± 0.05 | 0 ± 0 |  | 4.4 ± 0.49 | 2.7 ± 0.64 | 1.3 ± 0.42 |  | <0.001 | >0.05 | >0.05 |
| Myxococcota | 1.3 ± 0.04 | 2.6 ± 0.19 | 0 ± 0 |  | 1.3 ± 0.31 | 2.4 ± 0.14 | 2.4 ± 0.6 |  | <0.05 | >0.05 | >0.05 |
| Actinobacteriota | 5.4 ± 0.04 | 14.1 ± 1.61 | 1.8 ± 0.97 |  | 5.9 ± 0.41 | 16.1 ± 3.52 | 43.5 ± 3.21 |  | <0.05 | <0.05 | <0.05 |
| Gemmatimonadota | 3.1 ± 0.4 | 2.5 ± 0.39 | 0.1 ± 0.02 |  | 3.7 ± 0.25 | 2.7 ± 0.23 | 0.1 ± 0.06 |  | <0.001 | >0.05 | >0.05 |
| Desulfobacterota | 1 ± 0.16 | 0.5 ± 0.07 | 0 ± 0 |  | 0.8 ± 0.12 | 0.1 ± 0.05 | 0 ± 0 |  | <0.001 | <0.05 | >0.05 |
| Nitrospirota | 1 ± 0.17 | 0.5 ± 0.1 | 0 ± 0 |  | 1.2 ± 0.16 | 0.5 ± 0.09 | 0 ± 0 |  | <0.001 | >0.05 | >0.05 |
| RCP2-54 | 1.1 ± 0.21 | 0.4 ± 0.03 | 0 ± 0 |  | 0.7 ± 0.02 | 0.2 ± 0.09 | 0 ± 0 |  | <0.001 | >0.05 | >0.05 |
| Others | 3.9 ± 0.35 | 1.5 ± 0.29 | 0.1 ± 0.02 |  | 3.8 ± 0.87 | 0.9 ± 0.09 | 0.4 ± 0.23 |  | <0.001 | >0.05 | >0.05 |
| **Fungi** |  |  |  |  |  |  |  |  |  |  |  |
| Ascomycota | 92.4 ± 2.3 | 98.5 ± 0.44 | 94.3 ± 2.9 |  | 90.8 ± 4.4 | 97.7 ± 0.8 | 98.3 ± 0.97 |  | <0.05 | >0.05 | >0.05 |
| Basidiomycota | 2.5 ± 0.5 | 0.6 ± 0.06 | 0.1 ± 0.03 |  | 1.6 ± 0.15 | 0.3 ± 0.05 | 0.3 ± 0.17 |  | <0.001 | >0.05 | >0.05 |
| Mortierellomycota | 4.6 ± 2.5 | 1 ± 0.4 | 5.7 ± 2.8 |  | 7.5 ± 4.4 | 2 ± 0.74 | 1.6 ± 1.01 |  | >0.05 | >0.05 | >0.05 |
| Others | 0.6 ± 0.15 | 0.1 ± 0.02 | 0.1 ± 0.01 |  | 0.4 ± 0.08 | 0.1 ± 0.03 | 0.1 ± 0.01 |  | <0.001 | >0.05 | >0.05 |

**Table S4** Mantel test showing the Pearson’s correlation (r) of bacterial and fungal community structures with soil chemical properties based on Bray−Curtis distance.

|  | Bacteria | |  | Fungi | |
| --- | --- | --- | --- | --- | --- |
|  | Correlation coefficient | Adjusted p value |  | Correlation coefficient | Adjusted p value |
| pH | 0.250 | 0.079 |  | 0.423 | 0.032 |
| K | 0.933 | 0.005 |  | 0.441 | 0.051 |
| TN | 0.918 | 0.007 |  | 0.493 | 0.028 |
| TC | 0.935 | 0.005 |  | 0.511 | 0.028 |
| TP | 0.420 | 0.03 |  | 0.240 | 0.088 |
| NH_4_^+^ | 0.851 | 0.015 |  | 0.367 | 0.069 |
| EC | 0.486 | 0.015 |  | 0.291 | 0.070 |
| AP | 0.208 | 0.100 |  | 0.507 | 0.028 |
| NO_3_^-^ | 0.142 | 0.107 |  | 0.477 | 0.028 |
| CEC | 0.714 | 0.01 |  | 0.380 | 0.047 |

Exchangeable potassium (K), total nitrogen (TN), ammonium nitrogen (NH_4_^+^), available P_2_O_5_ (AP), soil organic matter (SOM), nitrate nitrogen (NO_3_^-^), electrical conductivity (EC), cation exchange capacity (CEC).
